# An analysis of indirect genetic effects on adult body weight of the Pacific white shrimp *Litopenaeus vannamei* at low rearing density

**DOI:** 10.1186/s12711-015-0164-y

**Published:** 2015-12-14

**Authors:** Sheng Luan, Kun Luo, Zhan Chai, Baoxiang Cao, Xianhong Meng, Xia Lu, Ning Liu, Shengyu Xu, Jie Kong

**Affiliations:** Key Laboratory for Sustainable Utilization of Marine Fisheries Resources, Ministry of Agriculture, Yellow Sea Fisheries Research Institute, Chinese Academy of Fishery Sciences, Nanjing Road 106, Qingdao, 266071 China; Function Laboratory for Marine Fisheries Science and Food Production Processes, Qingdao National Laboratory for Marine Science and Technology, No. 1 Wenhai Road, Aoshanwei Town, Jimo, Qingdao, China; Hainan Higene Aquaculture Technology Ltd., Wengtian Town, Wenchang, 571328 China

## Abstract

**Background:**

Our aim was to estimate the genetic parameters for the direct genetic effect (DGE) and indirect genetic effects (IGE) on adult body weight in the Pacific white shrimp. IGE is the heritable effect of an individual on the trait values of its group mates.

**Methods:**

To examine IGE on body weight, 4725 shrimp from 105 tagged families were tested in multiple small test groups (MSTG). Each family was separated into three groups (15 shrimp per group) that were randomly assigned to 105 concrete tanks with shrimp from two other families. To estimate breeding values, one large test group (OLTG) in a 300 m^2^ circular concrete tank was used for the communal rearing of 8398 individuals from 105 families. Body weight was measured after a growth-test period of more than 200 days. Variance components for body weight in the MSTG programs were estimated using an animal model excluding or including IGE whereas variance components in the OLTG programs were estimated using a conventional animal model that included only DGE. The correlation of DGE between MSTG and OLTG programs was estimated by a two-trait animal model that included or excluded IGE.

**Results:**

Heritability estimates for body weight from the conventional animal model in MSTG and OLTG programs were 0.26 ± 0.13 and 0.40 ± 0.06, respectively. The log likelihood ratio test revealed significant IGE on body weight. Total heritable variance was the sum of direct genetic variance (43.5 %), direct–indirect genetic covariance (2.1 %), and indirect genetic variance (54.4 %). It represented 73 % of the phenotypic variance and was more than two-fold greater than that (32 %) obtained by using a classical heritability model for body weight. Correlations of DGE on body weight between MSTG and OLTG programs were intermediate regardless of whether IGE were included or not in the model.

**Conclusions:**

Our results suggest that social interactions contributed to a large part of the heritable variation in body weight. Small and non-significant direct–indirect genetic correlations implied that neutral or slightly cooperative heritable interactions, rather than competition, were dominant in this population but this may be due to the low rearing density.

## Background

Social interactions between individuals have been extensively studied in animal and plant populations [1]. Such interactions may be due to a number of factors, including competition for limited resources (e.g., soil nutrients or food), social behaviors (e.g., aggression, social dominance, competitive ability, or helping behavior), or interactions between mothers and their offspring (maternal effects) [2]. Cannibalistic and aggressive behaviors were often reported in shrimp [3, 4], especially when the stocking density was high and feeding frequency was low. Such social interactions may affect the growth, survival and uniformity of the shrimp. It is difficult to improve the socially affected traits by classical selection methodologies that target only the direct genetic effect (DGE) of an individual on its own phenotype. Unfortunately, it is unrealistic to observe and record social behaviors among shrimp directly because of their small size and high stocking density as well as the complex water environment and time-consuming nature of the procedure. Therefore, in general, social interactions are ignored in most selective breeding programs. Ignoring the indirect genetic effect (IGE) of an individual on the phenotype of its group mates may result in a reversal of the direction of the selection response. In a population of Japanese quails, negative selection responses for 43-day body weight and mortality were obtained when selection of candidates was based on traditional estimated breeding values (EBV) [1]. Such negative selection responses occur because, in classical selection programs, the best individuals may have negative genetic effects on other individuals.

Extended quantitative genetic models have been developed to estimate DGE and IGE without the need for behavioral observation. Individual IGE can be predicted as a random effect in the mixed model using a test design that is capable of detecting a social effect. In the Atlantic cod *Gadus morhua*, the genetic parameters of DGE and IGE on growth and fin damage traits were estimated using the 3FAM design that consists of 100 small groups of three families [5]. IGE on harvest weight were estimated using an optimal design of multiple blocks of 11 families in the Nile tilapia *Oreochromis niloticus* [6, 7]. The total genetic variation that underlies a socially-affected trait can also be captured using such methods. For example, in the Atlantic cod, the heritable variance for length of the first dorsal fin that included variance of IGE was equal to 28.43 ± 6.60 and was more than three-fold greater than the additive genetic variance estimated by traditional methods (8.50 ± 0.147) [5]. Moreover, response to selection for socially affected traits can be increased using group selection or multilevel selection that takes IGE into account [8–13].

The Pacific white shrimp, *Litopenaeus vannamei*, is an important farmed penaeid shrimp that accounts for 42 % of the total shrimp production in the world [14]. Several classical selective breeding programs have been carried out and resulted in genetic gain for growth and disease resistance [15–19]. However, little is known regarding IGE in farmed shrimp. In this study, our aim was to estimate the genetic parameters of the DGE and IGE on adult body weight at a low rearing density in the Pacific white shrimp. Data from multiple small test groups (MSTG) was analyzed using both a classical animal model and an extended animal model that included DGE and IGE. Moreover, total heritable variance was estimated for each model and then compared. The correlation between the DGE obtained with MSTG and traditional EBV from one large test group (OLTG) was calculated to evaluate the accuracy of the predicted breeding value from MSTG.

## Methods

### Ethical statement

This research was approved by the Animal Care and Use committee in the Yellow Sea Fisheries Research Institute, Chinese Academy of Fishery Sciences; all shrimp were reared and tested at a low rearing density. All in vivo experiments in this study were performed in accordance with the Animal Research: Reporting in Vivo Experiments (ARRIVE) guidelines. Animals used in this study belonged to the Pacific white shrimp *L. vannamei* species, which originated from Latin American countries, including Brazil, Mexico, and Colombia. This shrimp was introduced into China for farming approximately in 1998 and is neither an endangered nor a protected species both in China and its native countries. All the experiments carried out in this study were in accordance with the Law of the People’s Republic of China on the Protection of Wildlife (http://www.china.org.cn/english/environment/34349.htm).

### Origin of the base population and selection procedure

The breeding program was performed at Hainan Higene Aquaculture Technology Ltd (longitude 110.952874, latitude 19.937534) in Wenchang City, Hainan Province, China, in 2012. The founder broodstocks were from eight improved batches that were introduced from different companies in the United States and Singapore. Individuals were checked for different virus strains using reverse transcriptase polymerase chain reaction and only the virus-free individuals were used. After isolation, each shrimp was individually tagged using numbered rings that were placed on one ocular peduncle. Broodstocks with a healthy appearance and mature gonads were chosen after one month of temporary rearing. The base population (G_0_ generation) was formed by an incomplete diallel cross experiment for eight strains. A total of 207 full-sib families (40 paternal half-sib families; 59 maternal half-sib families) from 187 sires and 174 dams were obtained by artificial insemination in 2012. These families were tagged using visible implant fluorescent elastomer (VIE) and communally reared in two 100 m^2^ tanks for the grow-out test.

Breeding candidates in generation G_0_ were ranked using a selection index [20], which included individual EBV for body weight, and the family EBV for survival. The mating schemes for the selection and control populations in generation G_1_ were created as follows. The selection population was created by mating primarily selected males and females with a high selection index (>100) from families with a high selection index (>100), with restrictions to control inbreeding (e.g., avoiding full-sib, half-sib and cousin mating), and maintaining a large effective population size [21]. The control population was composed of 10 to 20 full-sib families, which were produced from 20 to 30 breeding candidates of generation G_0_ by single-pair mating. A total of 105 families (10 paternal half-sib families; 26 maternal half-sib families) from 100 males and 91 females of generation G_0_ were selected to produce generation G_1_ (Table 1).

**Table 1:** Data structure and statistical description for body weight in generation G_1_ of *Litopenaeus vannamei*

Gen^a^	Sires	Dams	Full-sib families	Half-sib families	Test program	Sex	N1	N2	Mean^b^	Min^c^	Max^d^	SD^e^	CV^f^	Survival rate (%)
G_1_	100	91	105	33^g^	MSTG	All	4725	4282	26.88	6.40	42.50	4.38	16.29	90.62
						Male	/	2060	26.28	9.70	40.80	3.91	14.88	–
						Female	/	2222	27.44	6.40	42.50	4.71	17.16	–
					OLTG	All	8398	6515^h^	20.77	3.90	36.40	3.90	18.78	77.58
						Male	/	3187	20.39	6.80	32.40	3.46	16.97	–
						Female	/	3318	21.17	6.40	36.40	4.14	19.56	–

*MSTG* individuals from all families were tested in multiple small test groups (105 square concrete tanks of approximately 3 m^2^), *OLTG* individuals from all families were tested in one large test group (a circular concrete tank of 300 m^2^)

^a^Generation

^b^Mean body weight (g)

^c^Minimum body weight (g)

^d^Maximum body weight (g)

^e^Standard deviations for body weight (g)

^f^Coefficient of variation for body weight (%)

^g^Numbers of paternal and maternal half-sib families were equal to 10 and 26, respectively, but the number of half-sib families indicated is less than 36 because three full-sib families were identical between the paternal and maternal groups

^h^The sex of 10 individuals was not identified when measuring body weight at harvest, thus, the number of male and female shrimp was less than the total number of individuals with observations on body weight

### Programs to estimate the DGE and IGE

#### MSTG program

The 3FAM design was used to estimate the DGE and IGE on body weight in generation G_1_ [5, 22]. At the beginning of the experiment, each of the 105 families with 45 shrimp (4725 individuals) was tagged using VIE and equally divided into three groups when the average body weight was about 7 g. Next, each of the three groups from each family were randomly assigned to one of 105 concrete tanks (170 cm × 170 cm × 100 cm) of approximately 3 m^3^, with restrictions to control the coancestry coefficient (e.g., avoiding assigning half-sib and cousin families in the same tank) and decrease the coefficient of variation of body weight at tagging between the three families in a tank. In this design, each family was tested against six other families in three different tanks. Each tank contained 45 shrimp from three families that resulted in an average shrimp density of about 15 individuals/m^2^ at the beginning of the experiment. All shrimp were stocked in 105 tanks during five days, and were harvested over two days after a growth-test period of 217 days. The water exchange rate for each tank was about 20 to 30 % over 3 days. At the end of the experiment, the average density was about 14 individuals/m^3^.

#### OLTG program

To obtain the classical EBV and evaluate the genotype by environment interaction effects between the MSTG and OLTG programs, a circular concrete tank (300 m^2^) was used for communal rearing of 105 families as used in MSTG at generation G_1_. The progeny of each family were tagged using VIE and mixed for the grow-out test when the average body weight reached about 7 g. The stocking family size ranged from 14 to 90 individuals and was on average equal to 80. The 8398 individuals were stocked during eight days, and harvested over 6 days after a growth-test period of 216 days. The water exchange rate was only about 5 to 10 % over 7 days because this tank was equipped with a water recirculating system. At the end of the experiment, the average density was about 22 individuals/m^2^.

### Statistical analysis

Genetic analysis of body weight was performed using ASReml4 [23]. The data were first analyzed using a traditional animal model without IGE.

The following models were used.

For the MSTG program:

1 $$ \begin{aligned} y_{iklm} &= \mu + Sex_{l} + Age_{m} (Sex_{l} ) \hfill \\ & \quad + a_{{d_{i} }} + c_{m} + t_{k} + e_{iklm} ; \end{aligned}$$

For the OLTG program:

2 $$ y_{ilm} = \mu + Sex_{l} + TBW_{m} (Sex_{l} ) + a_{{d_{i} }} + e_{ilm} ; $$

For the MSTG program when both the DGE and IGE were included:

3 $$ y_{ijklm} = \mu + Sex_{l} + Age_{m} (Sex_{l} ) + a_{{d_{i} }} + \sum\limits_{i \ne j}^{n - 1} {a_{{s_{j} }} } + c_{m} + t_{k} + e_{ijklm} ; $$

where $y_{ijklm}$, $y_{iklm}$ or $y_{ilm}$ is the observed body weight of the *i*th shrimp; *μ* is the overall mean; $Sex_{l}$ is the fixed effect of the *l*th gender; ${\text{Age}}_{m} (Sex_{l} )$ is a linear covariate of age nested within the *l*th gender; $TBW_{m} (Sex_{l} )$ is a covariate of body weight at tagging, with the ‘spline’ function that uses the unique data values as the knot points in ASReml; $a_{{d_{i} }}$ is the DGE of the *i*th shrimp, $a_{d} \sim (0,{\mathbf{A}}\sigma_{{a_{d} }}^{2} )$, where **A** is the additive genetic relationship matrix among all shrimp included in generation G_1_ and its parents, and $\sigma_{{a_{d} }}^{2}$ is the variance of DGE; $\sum\nolimits_{i \ne j}^{n - 1} {a_{{s_{j} }} }$ is the sum of the IGE of n − 1 associates in the same group as the *i*th focal individual (n = 45), $a_{\text{s}} \sim (0,{\mathbf{A}}\sigma_{{a_{s} }}^{2} )$, where $\sigma_{{a_{\text{s}} }}^{2}$ is the variance of IGE; $c_{m}$ is the random effect common to the *m*th full-sib family, ${\text{c}}\sim (0,{\mathbf{I}}\sigma_{c}^{2} )$, which is a combination of the tank effect due to separate rearing of the full-sib families before stocking and one quarter of the non-additive (dominance) genetic effect common to full-sibs, where **I** is the identity matrix, and $\sigma_{c}^{2}$ is the variance of common environmental effect; $t_{k}$ is the random effect of the *k*th test tank, $t\sim (0,{\mathbf{I}}\sigma_{t}^{2} )$, $\sigma_{t}^{2}$ is the variance of the test tank effect; and $e_{ijklm}$, $e_{iklm}$ or $e_{ilm}$ is the random residual error of the *i*th individual, $e\sim (0,{\mathbf{I}}\sigma_{e}^{2} )$, where $\sigma_{e}^{2}$ is the residual variance.

The log likelihood ratio test showed that the *c* and *t* effects should have been included in Models 1 and 3. However, it did not converge when the *c* effect was included in Model 2. Therefore, body weight at tagging rather than age was fitted as the covariate to reduce the impact of common environmental effect in Model 2.

The total breeding value (TBV) was defined as the total heritable effect of an individual on trait values in the population, and was the breeding value of interest for response to selection [9].

4 $$ TBV_{i} = a_{{d_{i} }} + (n - 1)a_{{s_{i} }} . $$

Therefore, the variance of TBV ($\sigma_{TBV}^{2}$) was calculated as:

5 $$ \sigma_{TBV}^{2} = \sigma_{{a_{d} }}^{2} + 2(n - 1)\sigma_{{a_{ds} }} + (n - 1)^{2} \sigma_{{a_{s} }}^{2} , $$

where $\sigma_{{a_{ds} }}$ is the covariance between the DGE and IGE.

The phenotypic variance ($\sigma_{p}^{2}$) was calculated as:

6 $$ \sigma_{p}^{2} = \sigma_{{a_{d} }}^{2} + (n - 1)\sigma_{{a_{s} }}^{2} + \sigma_{c}^{2} + \sigma_{t}^{2} + \sigma_{e}^{2} . $$

In classical quantitative genetic theory, the heritability measure of the direct genetic variance relative to the phenotypic variance is calculated by $h^{2} = \sigma_{{a_{d} }}^{2} /\sigma_{p}^{2}$. By analogy, to express the heritable total variance to the phenotype variance, $T^{2}$ is introduced as the ratio of $\sigma_{TBV}^{2}$ to $\sigma_{p}^{2}$. In this study, $T^{2}$ only represents the heritable variance expressed on the scale of phenotypic variance among tested shrimp. Comparison of $T^{2}$ and $h^{2}$ gives a quick indication of the contribution of social effects to heritable variance.

The correlation of the DGE on body weight between the MSTG and OLTG programs was estimated by a bivariate animal model including or excluding IGE, where body weight in each program was treated as a separate trait. The *c* effect was not included in the models, and the residual covariance between the MSTG and OLTG programs was set to zero because some individuals differed between the two programs.

To account for heterogeneity due to the eight strains introduced, which may influence the genetic parameters for body weight and survival [24], eight genetic groups were included in the pedigree and considered using the !GROUP qualifier in ASReml.

## Results

### Descriptive statistics

The coefficients of variation for body weight for the MSTG and OLTG programs were equal to 16.29 and 18.78 %, respectively. The small tank size (3 vs. 300 m^2^) may have increased uniformity among individuals that could share the same environment and food. The mean and standard error of body weight for females were slightly greater than those observed for males. The average body weight (20.77 g) and survival rate (77.58 %) for the OLTG program were lower than those (26.88 g and 90.62 %) for the MSTG program. The water recirculating system that equipped the tank used in the OLTG program may have been responsible for this trend.

### Variance components and genetic parameters

Table 2 shows variance components and genetic parameters for body weight that were estimated using the conventional animal model (Models 1 and 2). Heritabilities of 0.26 ± 0.13 and 0.40 ± 0.06 for body weight were obtained for the MSTG and OLTG programs, respectively. The latter was likely biased upward because the *c* effect was not fitted in Model 2. For the MSTG program, the c^2^ for body weight was small (0.07 ± 0.06) and not significant (*P* < 0.05). The variance of the test tank explained 20.90 % of the phenotypic variance. The estimated correlation of the DGE of body weight between the MSTG and OLTG programs was equal to 0.71 ± 0.11.

**Table 2:** Genetic parameters estimated using two animal models for body weight in *Litopenaeus vannamei*

Parameters	Animal model without IGE		Animal model with IGE
	MSTG	OLTG	MSTG
$\sigma_{{a_{d} }}^{2}$	4.74 ± 2.55	6.26 ± 1.05	5.06 ± 2.46
$\sigma_{{a_{s} }}^{2}$	–	–	0.00395 ± 0.00282
$\sigma_{{a_{ds} }}$	–	–	0.00309 ± 0.0386
$\sigma_{c}^{2}$	1.29 ± 1.03	–	1.01 ± 0.96
$\sigma_{t}^{2}$	3.81 ± 0.61	–	1.46 ± 0.57
$\sigma_{TBV}^{2}$	–	–	11.63 ± 5.81
$\sigma_{e}^{2}$	8.34 ± 1.30	9.22 ± 0.58	8.18 ± 1.26
$\sigma_{p}^{2}$	18.19 ± 0.92	15.48 ± 0.57	15.88 ± 0.84
$T^{2}$	–	–	0.73 ± 0.37
$h^{2}$	0.26 ± 0.13	0.40 ± 0.06	0.32 ± 0.15
$c^{2}$	0.07 ± 0.06	–	0.06 ± 0.06
$r_{{a_{ds} }}$	–	–	0.02 ± 0.26
$r_{g}$	0.71 ± 0.11		0.68 ± 0.11

*IGE* indirect genetic effects, *MSTG* individuals from all families were tested in multiple small test groups (105 square concrete tanks of approximately 3 m^2^), *OLTG* individuals from all families were tested in one large test group (a circular concrete tank of 300 m^2^), $\sigma_{{a_{d} }}^{2}$ direct genetic variance, $\sigma_{{a_{s} }}^{2}$ indirect genetic variance, $\sigma_{{a_{ds} }}$ direct–indirect genetic covariance, $\sigma_{c}^{2}$ common environmental variance, $\sigma_{t}^{2}$ variance of the test tank effect, $\sigma_{TBV}^{2}$ variance of the total breeding value, $\sigma_{e}^{2}$ residual variance, $\sigma_{p}^{2}$ phenotypic variance, $T^{2}$ ratio of $\sigma_{TBV}^{2}$ to $\sigma_{p}^{2}$, $h^{2}$ heritability, $c^{2}$ common environmental coefficient, $r_{{a_{ds} }}$ correlation between the direct genetic effect (DGE) and IGE, $r_{g}$ genetic correlation on DGE between the MSTG and OLTG programs

For the MSTG program, IGE were found to be significant with the log likelihood ratio test (LRT) between Models 1 and 3 being equal to 86.58 (*P* < 0.001). Direct genetic variance was not significantly affected by the inclusion of IGE in Model 3 (Table 2). The small indirect genetic variance (0.00395 ± 0.00282) contributed substantially to the heritable variance. The estimated ratio (T^2^) of the total heritable variance over the phenotypic variance was 0.73 ± 0.37, which was more than two-fold that of classical heritability (0.32 ± 0.15). The estimated correlation between the DGE and IGE was small and not significant. The inclusion of IGE reduced estimated tank effects (Table 2) which indicated that tank effects partly originated from social interactions among individuals, rather than entirely from physical differences between tanks. Including IGE in the model only slightly reduced the estimated correlation of the DGE on body weight between the MSTG and OLTG programs (0.68 ± 0.11) (Table 2) because the values of the IGE were small compared to those of DGE. In this study, standard errors of the variance components and genetic parameters related to IGE were in general large because the amount of data was relatively limited.

## Discussion

In this study, the DGE and IGE on body weight at harvest were estimated for a shrimp *L. vannamei* population. The heritable variance including that of IGE represented 73 % of the phenotypic variance and was more than two-fold greater than that expected with a classical heritability analysis, which indicates that social interactions may contribute to a large part of the heritable variation. This result suggests that growth rate is strongly influenced by social interactions.

Genetic parameters for socially-affected traits have been estimated in several previous studies on natural or selected populations [25]. Social interactions have a substantial effect on the total heritable variance and explain 6 to 98 % of this variance [25]. For example, for Nile tilapia, it was reported that indirect genetic variance of body weight represented 48 % of the total heritable variance [6]. For the length of the first dorsal fin in farmed Atlantic cod, social interactions explained 45.4 % of the total heritable variation; direct genetic variance only accounted for 21.5 % of the total heritable variation [5]. However, no significant IGE were detected for growth traits in the same 6-week experiment.

In our study, we found small and non-significant direct–indirect genetic correlations, which suggested that heritable interactions in this population were not competitive, but were neutral or slightly cooperative. However, expression of social interactions may depend on the environmental conditions. The low rearing density and the strategy used to reduce the initial variation in body weight at tagging between families in the same tank may have reduced competition between individuals. High rearing density and restricted feeding may create strong competition among mates and result in a negative direct–indirect genetic correlation. Muir [1] reported a negative correlation (−0.56) in a Japanese quail population when feeding was restricted. A negative direct–indirect genetic correlation (−0.38 ± 0.19) was also found for body weight in the Nile tilapia, which resulted in the total heritable variance being smaller than the additive genetic variance [6].

For the MSTG program, the heritability estimated for body weight using the classical animal model was in line with the estimates reported for other shrimp selective breeding programs [18, 26, 27]. For the OLTG program, the heritability estimated for body weight was greater than those reported in the literature and was likely overestimated because the *c* effect was not included in Model 2 due to the problem of convergence. One explanation is that the OLTG dataset may not have possessed sufficient depth in the relationships between individuals for the mixed model to separate the common environmental effect because of weak genetic ties between the families (half-sib families accounted for only 31 % of families). However, the *c* effect was successfully estimated using the same pedigree in the MSTG dataset. In the MSTG program, individuals of each family were assigned to three different tanks and were communally reared with individuals of the other six families. Thus, compared to the OLTG program, in this case, the *c* effect from each full-sib family was not entirely confounded with the genetic effect and the tank effect due to this special data structure.

The estimated correlation of the DGE on body weight between the MSTG and OLTG programs was around 0.7 and this estimate was hardly affected by including IGE in the model. Other studies have reported higher (>0.80) genetic correlations of body weight between tank environments for shrimp populations [18, 27]. However, in our study, there were probably large environmental differences between the MSTG and OLTG programs. First, the tank size (3 m^2^) in the MSTG program was much smaller than that (300 m^2^) in the OLTG program. Second, the water quality in the MSTG program was better than that in the OLTG program because different rearing models were used (high water exchange model vs. low water exchange model). In addition, the fact that the *c* effect was not included in Model 2 for the OLTG program may have reduced the direct genetic correlation between the OLGT and MSTG programs. This is because the DGE in the OLTG program may include a component due to the *c* effect, which does not correlate with the DGE in the MSTG program.

The accuracy of estimated TBV can be improved using a structure consisting of repeated complete full-sib family groups [25]. However, the DGE and IGE will be completely confounded in such designs, and the associated genetic parameters cannot be estimated for this structure. For the DGE, the optimal design would be one (or a few) large tank(s) that contain all the families. However, in the 3FAM design, only one family was tested against six other families in three different tanks to estimate IGE [22]. Therefore, the accuracy of DGE would be decreased with MSTG compared to OLTG. Well-designed breeding programs are needed to improve the accuracy of the simultaneous estimations of direct and indirect genetic effects. DGE and IGE were probably more accurate in a design with blocks composed of multiple full-sib families per block. With the block design, each family was combined precisely once with each of the other families in the same block [6, 7].

Compared with large livestock and fish breeding programs (e.g., dairy cattle, pig and salmonids) [5, 22, 25], genetic evaluation of IGE may be more feasible in shrimp because shrimp have large full-sib family sizes, a small body size and short production cycle. IGE effects should therefore be considered in real shrimp breeding programs. In the shrimp breeding program used here, shrimp were communally reared in one or several large tanks for testing growth and other traits. Moreover, each family was backed up and reared in a separate tank to avoid the high death risk due to disease and management in the communal rearing population. Therefore, estimating IGE using the MSTG program is straightforward and feasible. Candidates can be selected based on their own performance in the communal population and their sib information from the population of IGE testing.

## Conclusions

In this study, we found a large total heritable variance, which suggested that social interactions may contribute to a large part of the heritable variation in body weight in *L. vannamei*. Genetic evaluation of IGE may be feasible and should be included in real shrimp breeding programs. The small and non-significant direct–indirect genetic correlation that we estimated implies that neutral or slightly cooperative heritable interactions rather than competition were dominant in this population at a low rearing density. In future experiments, we shall test for IGE at high rearing density and under restricted feeding to quantify direct–indirect genetic covariance.
